# Neuroprotective Effects of Meloxicam and Selegiline in Scopolamine-Induced Cognitive Impairment and Oxidative Stress

**DOI:** 10.1155/2012/974013

**Published:** 2012-03-22

**Authors:** Puchchakayala Goverdhan, Akina Sravanthi, Thati Mamatha

**Affiliations:** Centre for Neurodegenerative Disease and Aging Research Department of Pharmacology, Vaagdevi College of Pharmacy, Ramnagar, Hanamkonda, Warangal 506001, India

## Abstract

Alzheimer's disease (AD) is a progressive neurodegenerative disorder characterized by a gradual decline in memory associated with shrinkage of brain tissue, with localized loss of neurons mainly in the hippocampus and basal forebrain, with diminished level of central cholinergic neurotransmitter-acetylcholine and also reported to be associated with accumulation of ubiquitinated proteins in neuronal inclusions and also with signs of inflammation. In these disorders, the abnormal protein aggregates may themselves trigger the expression of inflammatory mediators, such as cyclooxygenase 2 (COX-2). In the present study, the effects of Meloxicam, Selegiline, and coadministration of these drugs on scopolamine-induced learning and memory impairments in mice were investigated. Rectangular maze test, Morris water maze test, Locomotor activity, and Pole climbing test were conducted to evaluate the learning and memory parameters. Various biochemical parameters such as acetylcholinesterase(AChE), TBARS assay, catalase activity, and DPPH assay were also assessed. The present study demonstrates that Meloxicam, Selegiline, and co-administration of these test drugs had potential therapeutic effects on improving the antiamnesic activity in mice through inhibiting lipid peroxidation, augmenting endogenous antioxidant enzymes, and decreasing acetylcholinesterase activity in brain. The memory enhancing capacity of the drugs was very significant when compared to disease control (*P* < 0.001).

## 1. Introduction

Alzheimer's disease (AD) is a progressive neurodegenerative brain disorder that is slow in onset but leads to dementia, unusual behavior, personality changes, and ultimately death [1]. AD is characterized by the presence of excessive amounts of neuritic plaques containing amyloid *β* protein and abnormal tau protein filaments in the form of neurofibrillary tangles. Loss of cholinergic cells, particularly in the basal forebrain, is accompanied by loss of the neurotransmitter acetylcholine [2]. A decrease in acetyl choline in the brain of patients with AD appears to be a critical element in producing dementia [3]. AChE inhibitors from general chemical classes such as physostigmine, tacrine, galantamine, and heptylphysostigmine have been tested for the symptomatic treatment of AD [4]. However, nonselectivity of these drugs, their limited efficacy, poor bioavailability, adverse cholinergic side effects in the periphery, narrow therapeutic ranges, and hepatotoxicity are among the several limitations to their therapeutic success [5]. Therefore, it is worthwhile to explore the utility of other existing medicines for the treatment of various cognitive disorders [6].

 Scopolamine, a muscarinic cholinergic receptor antagonist, has been widely adopted to study cognitive deficits in experimental animals. After intraperitoneal (i.p.) injection of scopolamine, the cholinergic neurotransmission was blockaded, leading to cholinergic dysfunction and impaired cognition in rats [7]. Recently, it has been reported that memory impairment induced by scopolamine in rats is associated with altered brain oxidative stress status [8]. Therefore, rats with scopolamine-induced memory deficits were used as an animal model for screening antidementia drugs [9].

 Oxidative stress is also one of the affecting factors in AD, so several antioxidants have been studied for the reduction of oxidative stress occurring during Alzheimer's disease [10, 11]. One of the mechanisms by which the abnormal accumulation of ubiquitinated proteins may mediate neurodegeneration is by triggering an inflammatory response. Inflammation is a defense reaction against diverse insults, intended to remove damaging agents and to inhibit their detrimental effects [12]. Those agents were found to increase neuronal levels of cyclooxygenase 2 (COX-2) suggesting that the production of such inflammatory mediators can be triggered by the intracellular accumulation of abnormal proteins [13]. Nonsteroidal anti-inflammatory drugs (NSAIDs) are the group of drugs which effectively interfere with the cyclooxygenase pathway which is involved in generation of oxidative free radicals. In rheumatoid arthritis, NSAIDs have showed improvement in the circulating antioxidant status on daily dosing treatment [14, 15].

 For that purpose, meloxicam (an enolic derived NSAID) has been taken as reference drug by basing on the possession of significant anti-inflammatory activity as well as antioxidant property [16]. It has preferential inhibitory activity against the inducible cyclooxygenase-2 isoform, over the constitutive isoform cyclooxygenase-1. Therefore, meloxicam and other COX-2 selective inhibitors are promoted for their safer profile of side effects.

 Selegiline (L-deprenyl), an irreversible inhibitor of monoamine oxidase-B (MAO-B), a therapeutic agent of Parkinson's disease, is known to have neuroprotective properties that may involve its regulatory effects on antioxidant enzymes. In addition, selegiline may act as an antioxidant in neurons and protect against glutamate-receptor-mediated toxicity. Studies of selegiline on aged male laboratory animals have showed delayed cognitive impairment and behavioral deterioration when compared with control animals [17].

The main purpose of the present study was to investigate the synergistic action of meloxicam and selegiline in scopolamine-induced Alzheimer's disease model.

## 2. Materials and Methods

### 2.1. Animals

 Swiss mice of male sex weighing 20–25 g were used in the present study. They had free access to food and water and were maintained under standard laboratory conditions with alternating light and dark cycles of 12 h each. They were acclimatized to laboratory conditions for 2 days before behavioral studies. All the readings were taken during the same time of the day, that is, between 10 am and 2 pm. The Institution Animals Ethics Committee (IAEC) had approved the experimental protocol, and care of animals was taken as per guidelines of CPCSEA, Department of Animal Welfare, and Government of India [18].

### 2.2. Drugs

 Scopolamine (Cadila Healthcare pvt. Ltd), Selegiline (INTAS pharmaceuticals), and Donepezil (Alkem laboratories Ltd.) were purchased. Meloxicam was gifted by Dr. Reddy's Labarotaries. Scopolamine and selegiline were diluted with distilled water.

### 2.3. Experimental Design

The animals (*n* = 36) were divided into six different groups of 6 animals per each group. Scopolamine (1.4 mg/kg) as a disease inducer was administered to all groups through intraperitoneal (i.p) route after drugs administration to all the groups except normal control group. The same procedure was carried out for 9 days (see Table 1).

### 2.4. Behavioural Tests

 All the animals were trained for 2 days before drugs administration.

#### 2.4.1. Rectangular Maze Test

 Assessment of learning and memory can be effectively done by this method. The maze consists of completely enclosed rectangular box with an entry and reward chamber appended at opposite ends. The box is partitioned with wooden slats into blind passages leaving just twisting corridor leading from the entry to the reward chamber. Animals were trained prior to the experiment by familiarizing with the rectangular maze for a period of 10 min for 2 h. Well-trained animals were taken for the experiment. Transfer latency (time taken to reach the reward chamber) was recorded. For each animal, four readings were taken and the average is taken as learning score (transfer latency) for that animal. Lower scores of assessment indicate efficient learning while higher scores indicate poor learning in animals. The time taken by the animals to reach the reward chamber from the entry chamber was noted on day 1, 3, 5, 7, and 9 [19].

#### 2.4.2. Morris Water Maze Test

Morris water maze was used to assess learning and memory in experimental mice. There are several advantages of Morris water maze over other models of learning and memory including absence of motivational stimuli such as food and water deprivation, electrical stimulations, and buzzer sounds [20, 21]. Briefly, it consists of a circular water tank, filled with opaque water, and one centimeter submerged platform. First, animals were trained to locate the platform. During acquisition, trial escape latency time (ELT), time measure to locate the hidden platform, was noted as an index of acquisition. Each animal was subjected to the four acquisition trials per day for 4 consecutive days. The time spent by the animal, searching for the missing platform in target quadrant Q2 with respect to other quadrant (Q1, Q3, and Q4) on 5th day, was noted as an index of retrieval. For studying the effect of drug on acquisition, the drug solution was administered before acquisition trial [22].

#### 2.4.3. Locomotor Activity

Locomotor activity is influenced by most of the CNS drugs in both man and animals. The locomotor activity of drug can be studied using actophotometer which operates on photoelectric cells which are connected in circuit with a counter when the beam of light falling on photocell is cut off by the animal, then a count is recorded. Animals are placed individually in the activity cage for 10 min and the activity was monitored. The test is done before 30 min and after the drug administration. The photo cell count is noted and decrease or increase in locomotor activity is calculated [20].

#### 2.4.4. Pole Climbing Test

When an electrical stimulus is given to animal, it tries to escape from it and move to the near safe place. This equipment is designed in such a way to climb the pole when stimulus is generated. Prior to the experiment, animals were trained. Training and testing is conducted in a 25 × 25 × 40 cm chamber that is enclosed in a dimly light, sound attenuated box. Scrambled shock is delivered to the grid floor of the chamber. A smooth stainless steel pole, 2.5 cm in diameter, is suspended by a counter balance weight through a hole in the upper centre of the chamber. A micro switch is activated when the pole is pulled down by 3 mm. With weight greater than 200 gm. A response is recorded when a mice jumps on the pole and activates micro switch. The activation of light and speaker together is used as conditioned stimulus. Each animal was placed six times per day [20].

### 2.5. Histopathological Studies

After 8-day treatment, the brains of different groups were perfusion-fixed with 4% paraformaldehyde in 0.1 M phosphate buffer. The brains were removed and postfixed in the same fixative overnight at 48°C. The brains were then routinely embedded in paraffin and stained with Hematoxylin-Eosin. The hippocampal lesions were assessed microscopically at 40 magnification [23].

### 2.6. Dissection and Homogenization

 On day 9, after behavioral assessments, animals were scarified by cervical dislocation. The brains were removed. Each brain was separately put on ice and rinsed with ice-cold isotonic saline. A (10% w/v) homogenate was prepared in 0.1 M phosphate buffer (pH 7.4). The homogenate was centrifuged at 3000 rpm for 15 minutes and aliquots of supernatant were separated and used for biochemical estimation [23].

### 2.7. Biochemical Tests

#### 2.7.1. AchE Estimation

The cholinergic marker, acetylcholinesterase, was estimated in the whole brain according to the method of Ellman method. Ellman's reagent is 5, 5′-dithiobis(2-nitrobenzoate) and it is also abbreviated as DTNB. This homogenate was incubated for 5 min with 2.7 mL of phosphate buffer and 0.1 mL of DTNB. Then, 0.1 mL of freshly prepared acetylthiocholine iodide (pH 8) was added and the absorbance was read at 412 nm [24, 25].

#### 2.7.2. Thiobarbituric Acid Reactive Substances (TBARS) Assay

This assay is used to determine the lipid peroxidation. Aliquots of 0.5 mL distilled water were added with1 mL of 10% trichloroacetic acid and were added with 0.5 mL of brain tissue homogenate. This is centrifuged at 3000 rpm for 10 min. To the 0.2 mL supernatant, 0.1 mL thiobarbituric acid (0.375%) was added. Total solution is placed in water bath at 80°c for 40 min and cooled at room temperature. Absorbance was read at 532 nm [26].

#### 2.7.3. Catalase Activity

Catalase activity was assessed by the method of Luck [27], wherein the breakdown of hydrogen peroxide is measured. In this 3 mL of H_2_O_2_ phosphate buffer was added to 0.05 mL of the supernatant of the tissue homogenate. The absorbance was recorded at 240 nm using Perkin Elmer Lambda 20 spectrophotometer. The results were expressed as micromoles of H_2_O_2 _decomposed per minute per mg protein [25].

#### 2.7.4. DPPH (2,2-Diphenyl-1-picrylhydrazyl) Assay

In this, measurement is made from the bleaching of purple-coloured methanol solution of DPPH. To the 1000 *μ*L of diverse conc. of the sample, 4 mL of 0.004% methanolic solution of DPPH was added. After 30 min incubation, absorbance was read at 517 nm. Inhibition of free radical by DPPH in % was calculated in the following way:

(1) $$\%=\left(A_{\text{ blank}}-A_{\text{ sample}}/A_{\text{ blank}}\right)\times 100,$$

*A* _blank_: absorbance of control reaction. *A *_sample_: absorbance of test sample. Values of inhibition were calculated [26].

### 2.8. Statistical Analysis

 The statistical analysis of data was done by the one way analysis of variance (ANOVA) followed by the Dunnett's test. The probability level less than 0.05 was considered as significant. Results were expressed as mean ± SD.

## 3. Results

### 3.1. Behavioural Tests

#### 3.1.1. Rectangular Maze Test

**Figure 1:**
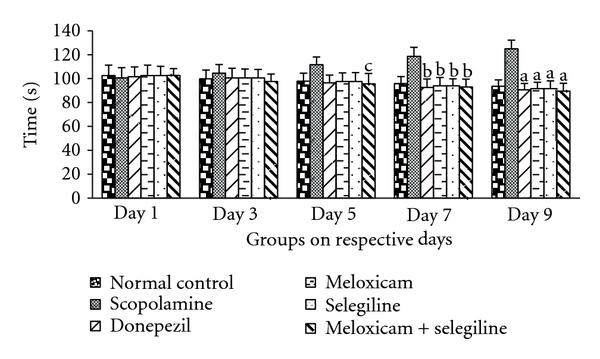
Rectangular maze test. Effect of meloxicam and selegiline on latency time compared to the disease control group. (Mean ± SD, *n* = 6). Graph showing mean ± SD of latency time in seconds. ^a^ *P* < 0.001, ^b^ *P* < 0.01, ^c^ *P* < 0.05 compared with corresponding values of disease control.

The activity of meloxicam and selegiline was evaluated using rectangular maze. The mice in all treatment groups except scopolamine-treated group showed lower transfer latency on 7th day and 9th day compared to 5th day of the same group as well as with the scopolamine group which was given in Figure 1. This indicates memory enhancing capacity of the meloxicam and selegiline. Donepezil (5 mg/kg) treated for successive 8 days acts as positive control, possessed significant (*P* < 0.05) decrease in transfer latency when compared to normal control and disease control (scopolamine) using Dunnet's test.

#### 3.1.2. Morris Water Maze Test

**Figure 2:**
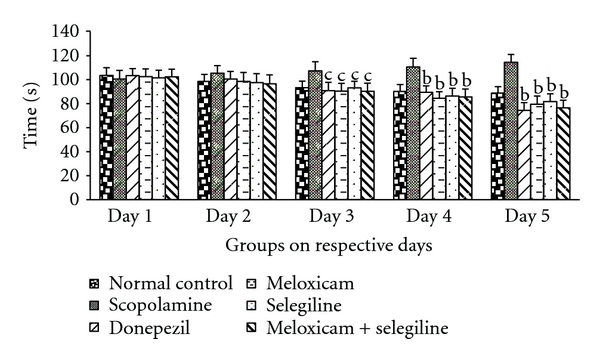
Morris water maze test. Effect of meloxicam and selegiline on latency time compared to the disease control group. (Mean ± SD, *n* = 6). Graph showing mean ± SD of latency time in seconds. ^a^ *P* < 0.001, ^b^ *P* < 0.01, ^c^ *P* < 0.05 compared with corresponding values of disease control.

The activity of meloxicam and selegiline wAS evaluated using Morris water maze. The mice treatment groups except scopolamine-treated group showed significant transfer latency on 4th day with platform and on 5th day without platform which was given in Figure 2. This indicates memory enhancing capacity of the meloxicam and selegiline. Donepezil (5 mg/kg) treated for successive 8 days acts as positive control, possessed significant (*P* < 0.05) decrease in transfer latency when compared to disease control (scopolamine) using dunnet's test.

#### 3.1.3. Locomotor Activity

**Figure 3:**
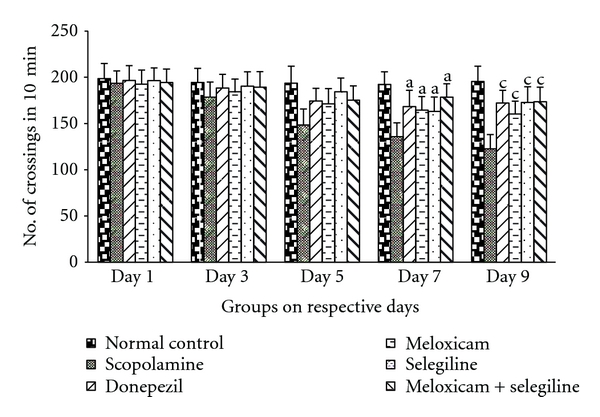
Locomotor activity. Effect of meloxicam and selegiline on latency time compared to the disease control group. (Mean ± SD, *n* = 6). Graph showing mean ± SD of latency time in seconds. ^a^ *P* < 0.001, ^b^ *P* < 0.01, ^c^ *P* < 0.05 compared with corresponding values of disease control.

The activity of meloxicam and selegiline was evaluated using photoactometer. The mice showed significant transfer latency on 7th day compared to the 9th day in all treatment groups except scopolamine-treated group which was given in Figure 3. This Donepezil (5 mg/kg) treated successive 8 days acts as positive control, possessed significant (*P* < 0.05) decrease in number of crossings which is comparable to the other treatment groups.

#### 3.1.4. Pole Climbing Test

 The values show that there was a significant difference that has been observed on days 7 and 9 compared to the 1, 3, and 5. Scopolamine-treated group took more time whereas the control and drug-treated groups showed less time to reach the pole in pole climbing apparatus. The results showed that synergistic action of meloxicam and selegiline was significant (*P* < 0.05) and is comparable to the standard drug (donepezil).

### 3.2. Biochemical Tests

#### 3.2.1. AchE Estimation

Scopolamine treatment significantly increased the brain AchE level compared to control group (Figure 5). Standard drug (donepezil) and test drugs (meloxicam, selegiline) treatment significantly inhibited the brain AchE level compared to their corresponding scopolamine-treated groups.

#### 3.2.2. TBARS Assay

 Scopolamine treatment significantly increased the brain MDA level compared to control group (Figure 6). Standard drug (donepezil) and test drugs (meloxicam,selegiline) treatment significantly (*P* < 0.05) decreased brain MDA level compared to their corresponding scopolamine treated groups.

#### 3.2.3. Catalase Activity

 Catalase levels were decreased in scopolamine-treated groups compared to the normal control group (Figure 7). Significant (*P* < 0.05) difference has been found in drug-treated groups. Synergistic effect was observed which is comparable with the standard group than individual drug-treated groups.

#### 3.2.4. DPPH Assay

Antioxidant levels were decreased in scopolamine-treated group compared to the control group (Figure 8). Drug-treated groups showed significant (*P* < 0.05) difference compared to the disease control group.

### 3.3. Histopathological Studies

 From Figure 9, it is clearly visible that in disease control group the degenerated cells are more compared to other groups. This will be indicated by the gaps in slides. The drug-treated groups are in between the normal control and disease control groups. The combination group is mostly near to the control group compared to the individual drug-treated groups.

## 4. Discussion

 The scopolamine amnesia test is widely used as primary screening test for so-called anti-Alzheimer drugs [24].

 There recently has been an increased appreciation of the role that inflammation plays in the pathogenesis of Alzheimer's disease that has arisen principally from epidemiological studies showing a dramatic effect of long-term NSAID treatment on Alzheimer's disease risk. However, the molecuar mechanisms by which NSAIDs intervene in the pathological processes that underlie cognitive decline and neuronal loss remain unclear [28, 29].

 Recently, many studies reported that memory impairment in the scopolamine-induced animal model is associated with increased oxidative stress within the brain [8, 30, 31]. Oxidative stress is the cytotoxic consequence of oxyradical and oxidant formation and the reaction with cellular constituents. Reactive oxidative species (ROS) are generated continuously in nervous system during normal metabolism and neuronal activity. The nervous system is particularly vulnerable to the deleterious effects of ROS. Because the brain has a high consumption of oxygen, large amount of polyunsaturated fatty acids (PUFAs), high contents of free ions, and low levels of antioxidants defense were compared to other organs **[**32**]**. Increased MDA level as one of the ROS has been shown to be an important marker for in vivo lipid peroxidation.

**Figure 4:**
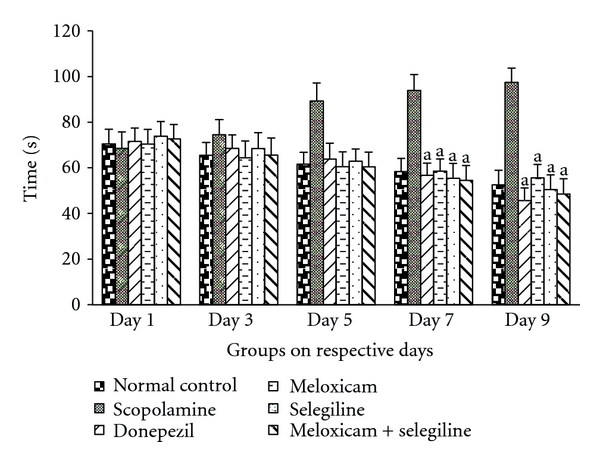
Pole climbing test: Effect of meloxicam and selegiline on latency time levels compared to the disease control group (Mean ± SD, *n* = 6). Graph showing mean ± SD of latency time in seconds. ^a^ *P* < 0.001, ^b^ *P* < 0.01, ^c^ *P* < 0.05 compared with corresponding values of disease control.

**Figure 5:**
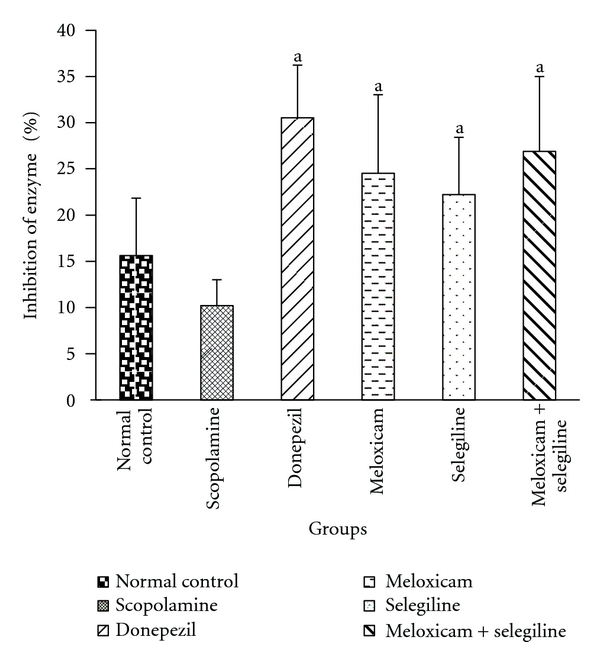
AchE estimation. Effect of meloxicam and selegiline on AchE levels compared to the disease control group. (Mean ± SD, *n* = 6). Graph showing mean ± SD of % inhibition of AchE enzyme. ^a^ *P* < 0.001 compared with corresponding values of disease control.

**Figure 6:**
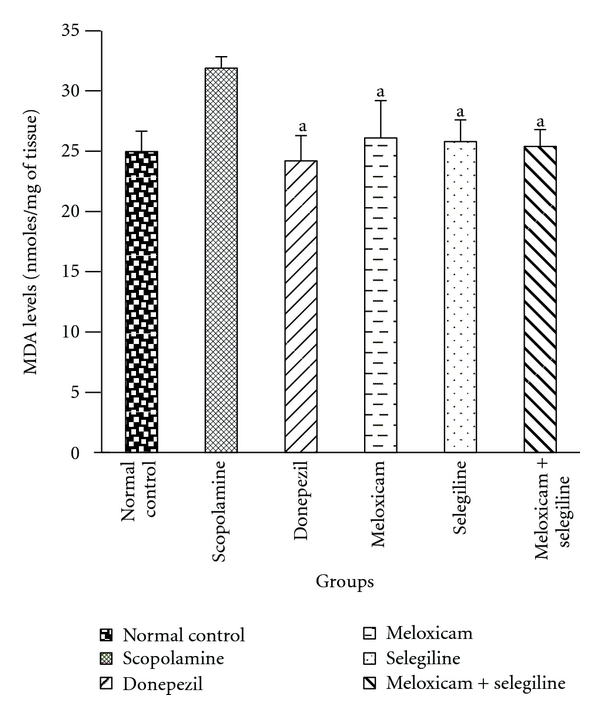
TBARS assay. Effect of meloxicam and selegiline on malondialdehyde levels compared to the disease control group. (Mean ± SD, *n* = 6). Graph showing mean ± SD of malondialdehyde levels. ^a^ *P* < 0.001 compared with corresponding values of disease control.

**Figure 7:**
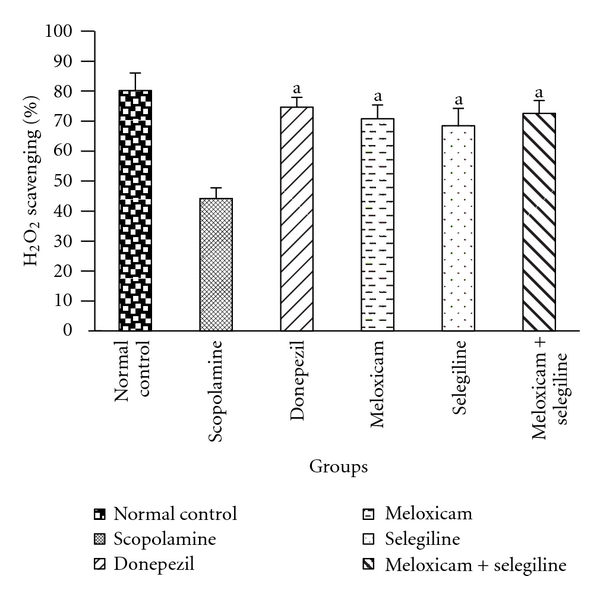
Catalase activity. Effect of meloxicam and selegiline on catalase activity compared to the disease control group. (Mean ± SD, *n* = 6). Graph showing mean ± SD of %H_2_O_2_ scavenging activity. ^a^ *P* < 0.001 compared with corresponding values of disease control.

**Figure 8:**
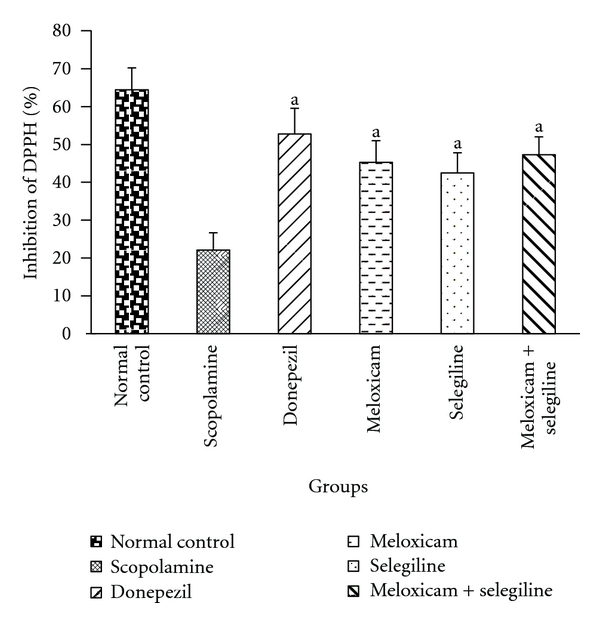
DPPH assay. Effect of meloxicam and selegiline on inhibition of DPPH compared to the disease control group (Mean ± SD, *n* = 6). Graph showing mean ± SD of % inhibition of DPPH. *P* < 0.001 compared with corresponding values of disease control.

**Figure 9:**
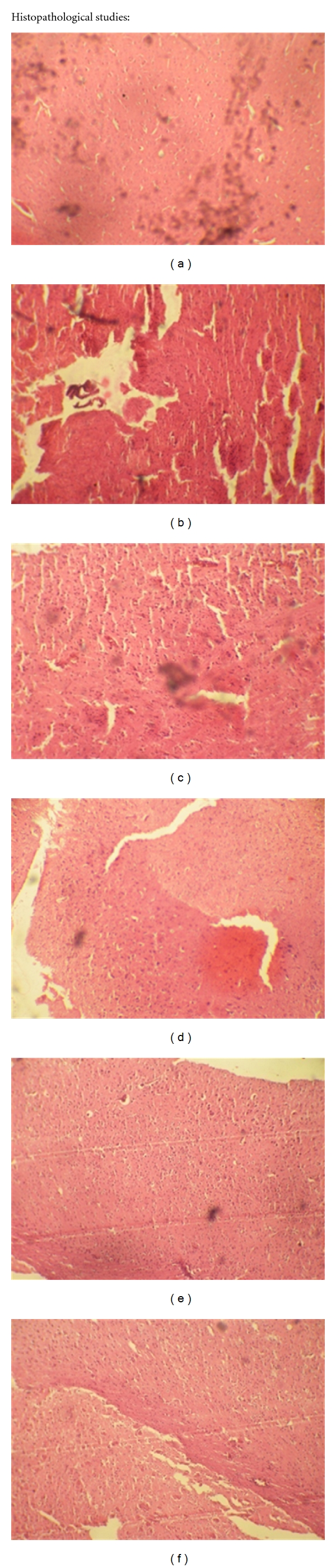
Histopathological studies. These Figures (a), (b), (c), (d), (e), and (f) are normal control, scopolamine (disease control), donepezil (standard), meloxicam, selegiline, and meloxicam + selegiline, respectively, representing the histological sections of the brain tissue showing neurological lesions.

 From the behavioral test, that is, rectangular maze test and Morris water maze test, it is clearly seen that there was a general decrease in the transfer latency in all treated groups compared to the scopolamine-treated group. The memory loss effect of scopolamine is more prominent compared to the control group. In comparison with Donepezil, the drug-treated groups had almost equal performance which indicates synergistic effect of meloxicam and selegiline against memory loss. Meanwhile locomotor activity and pole climbing avoidance tests are done which also indicate the leaning ability (Figure 4).

 The major antioxidant and oxidative free radical scavenging enzymes like glutathione, SOD, and catalase play an important role to reduce oxidative stress in brain. In this study, from the DPPH assay antioxidant levels are estimated. These enzyme levels are decreased in the scopolamine-treated group compared to the control group. The enzyme levels are almost equal in combination group and the standard group. Individual groups are showing less than standard group. It supports the antioxidant action of drugs.

 In the present study rats after scopolamine treatment showed a significant increase in the brain levels of malondialdehyde, which is the measure of lipid peroxidation and free radical generation. In the drug-treated groups, there is a significant decrease in the levels of malondialdehyde which is nearly equal to the standard group. From the results, it is clear that the anti-inflammatory activity of meloxicam decreases the disease progression. The antioxidant activity of selegiline is clear from the biochemical tests, which includes the estimation of antioxidant enzymes.

## 5. Conclusion

 In conclusion, the present study demonstrates that Meloxicam, Selegiline, and co-administration of these test drugs had potential therapeutic effects on improving the antiamnesic activity in mice through inhibiting lipid peroxidation, augmenting endogenous antioxidant enzymes, and decreasing acetylcholinesterase (AChE) activity in brain.

## Figures and Tables

**Table 1:** 

Group-I	Control	Vehicle (0.1% CMC).
Group-II	Disease control	Scopolamine (1.4 mg/kg) i.p.
Group-III	Standard	Donepezil (5 mg/kg) oral + Scopolamine (1.4 mg/kg) i.p.
Group-IV	Test-I	Meloxicam (5.2 mg/kg) oral + Scopolamine (1.4 mg/kg) i.p.
Group-V	Test-II	Selegiline (0.49 mg/kg) p.o. + Scopolamine (1.4 mg/kg) i.p.
Group-VI	Test-III	Meloxicam (5.2 mg/kg) oral + Selegiline (0.49 mg/kg) oral + Scopolamine (1.4 mg/kg) i.p.
